# Experimental dataset for effective bending stiffness and load-carrying capacity of CLT-concrete composite floor systems

**DOI:** 10.1016/j.dib.2025.112170

**Published:** 2025-10-16

**Authors:** Md Shahnewaz, Robert Jackson, Thomas Tannert

**Affiliations:** a Fast + Epp, Vancouver, British Columbia, Canada; b School of Engineering, University of Northern British Columbia, Prince George, Canada

**Keywords:** Cross-laminated timber, Timber floor, Timber connections, Composite floor

## Abstract

This article presents the dataset from experimental investigations on cross-laminated timber (CLT) concrete composite (TCC) floor systems. The data was collected at the University of Northern British Columbia, BC, Canada, for project-specific design decisions for “Limberlost Place”, a mass timber building for George Brown College, in Toronto, Canada designed by Fast + Epp, Vancouver, BC, Canada. The TCC systems comprised of 245 mm thick, 7-ply CLT panels with 150 mm concrete topping. A total of 18 small-scale shear connections, and 24 half-scale (4.6 m long) and 9 full-scale (9.2 m long) floors were tested. The dataset provides information on the effective bending stiffness and load-carrying capacity of long span beamless CLT-concrete floor systems. This data will also serve as essential inputs for engineers and researchers in modelling and design processes.

Specifications Table

**Table:** 

Subject	Civil and Structural Engineering
Specific subject area	Shear capacity, bending capacity, floor stiffness.
Data format	Raw, Analyzed
Type of data	Table, Image, Graph, Figure
Data collection	Dataset #1 was collected from a test set-up consisted of a compression load frame and a Linear Variable Differential Transformer (LVDT) sensor attached to each specimen. Dataset #2 contains deflections, interface slips, and edge warping measured for half-scale tests. Dataset #3 contains deflections, interface slips, and edge warping measured for full-scale tests. The data were collected after applying loads at one-third points using two 500 kN and two 250 kN actuators and recording using a total of 17 sensors (LVDT and string pots) were attached to the half-scale specimens and 20 sensors (LVDT and string pots) were attached to the full-scale specimens.
Data source location	Institution: University of Northern British Columbia City/Town/Region: Prince George Country: Canada Latitude and longitude for collected samples/data: 53.91559891860918, −122.74320614814793
Data accessibility	Repository name: Mendeley Data identification number: 10.17632/9j622zfd4s.1 Direct URL to data: https://data.mendeley.com/datasets/9j622zfd4s/1
Related research article	Shahnewaz, M., Jackson, and Tannert, T. (2023). Reinforced Cross-Laminated Timber-Concrete Composite Floor Systems. Engineering Structures 291(2023), 116,395. [1]

## 1 Value of the Data

• These datasets provide stiffness, shear, and bending capacities for long-span timber-concrete composite floor systems.
• The datasets can be used by Engineers and Architects for designing composite systems and by researchers for modelling purposes.
• These datasets can be used for the validation of analytical and numerical studies on timber-composite systems. The datasets can be reused and expanded for parametric study.

## 2 Background

The potential use of engineered wood products, such as CLT, for larger and non-residential structures with longer floor spans can significantly contribute to reducing global carbon emissions. The 10-storey Limberlost Place, located on George Brown College’s Toronto waterfront campus, holds the distinction of being Ontario's first tall timber institutional building, blending sustainable design and structural innovation. A large-span beamless CLT TCC ‘slab band’ system was developed, accompanied by perpendicular CLT infill panels, supported on glulam columns, enabling architectural flexibility and unobstructed mechanical distribution. The TCC slab band, with its reduced need for glulam beams, not only enhances spatial efficiency but also proves to be cost-effective through the application of the kerf-plate shear connector. This connector, evaluated through extensive testing, was the most economical option when compared to existing proprietary solutions. The dataset from this research will serve as a valuable tool in assessing the performance of long span timber, providing information on the effective bending stiffness and load-carrying capacity, aiding engineers and architects in designing long-span timber floors for ultimate limit state and serviceability limit state demands (i.e., deflections and vibration). Additionally, the data will be a valuable source for the research community for further development of other long-span timber floors.

## 3 Data Description

The descriptions of connectors and three datasets are illustrated in Table 1, Table 2, respectively and also available in previous publications [1,2]. TCC connectors used for dataset #2 and #3 are described in Table 3. The dataset is available on the Mendeley repository system under the title ‘Experimental dataset for cross-laminated timber-concrete composite floor systems’ [3].

**Table 1:** Test series overview (Data Set #1).

Series	Description	Test Type	#of tests	*length*	*width*	*thickness*
				[mm]	[mm]	[mm]
S1-A	35 mm connector depth	Shear	6	1000	300	395
S1-B	70 mm connector depth	Shear	6	1000	300	395
S1-C	90 mm connector depth	Shear	6	1000	300	395

**Table 2:** Test series overview (Data Set #2 and Data Set #3).

Series		Test ID	Screw Reinf.	Connector Type	Test Type	#of tests	Dimensions [mm]
Data set #2 half-span	S2	S2-UR	none	none	Bending	2	*L* = 5030
		S2-HR	low			2	*L_o_* = 4200
		S2-FR	high			2	*b_c_* = 2200
	S3	S3-UR-A	none	Type A	Bending	2	*b_t_* = 2400
		S3-HR-A	low			2	*a* = 1285
		S3-FR-A	high			2	
	S4	S4-UR-B	none	Type B	Bending	2	
		S4-HR-B	low			2	
		S4-FR-B	high			2	
	S5	S5-UR-C	none	Type C	Bending	2	
		S5-HR-C	low			2	
		S5-FR-C	high			2	
Data set #3 full-span	S6	S6-HR-A	low	Type A	Bending	2	*L* = 9630
		S6-HR-B		Type B		2	*L_o_* = 9200
		S6-HR-C		Type C		2	*b_c_* = 2200
	S7	S7-HR-A	low	Type A	Torsion	1	*b_t_* = 2400
		S7-HR-B		Type B		1	*a* = 3115
		S7-HR-C		Type C		1	

Note: *L* = total length of specimen, *L_o_* = span length of specimen between supports, *b_c_* = width of concrete, *b_t_* = width of CLT, *a* = distance from loading to support.

**Table 3:** Properties TCC shear connectors.

TCC Type	Description	Installation
Type A	STS ø11×250 mm fully threaded	Installed at 45° to grain
Type B	75×2100 mm steel plates	Installed into 7 mm wide saw kerf perpendicular to the span
Type C	90 mm deep HBV mesh	Glued into 3 mm wide saw kerf parallel to the span

**Fig. 1:**
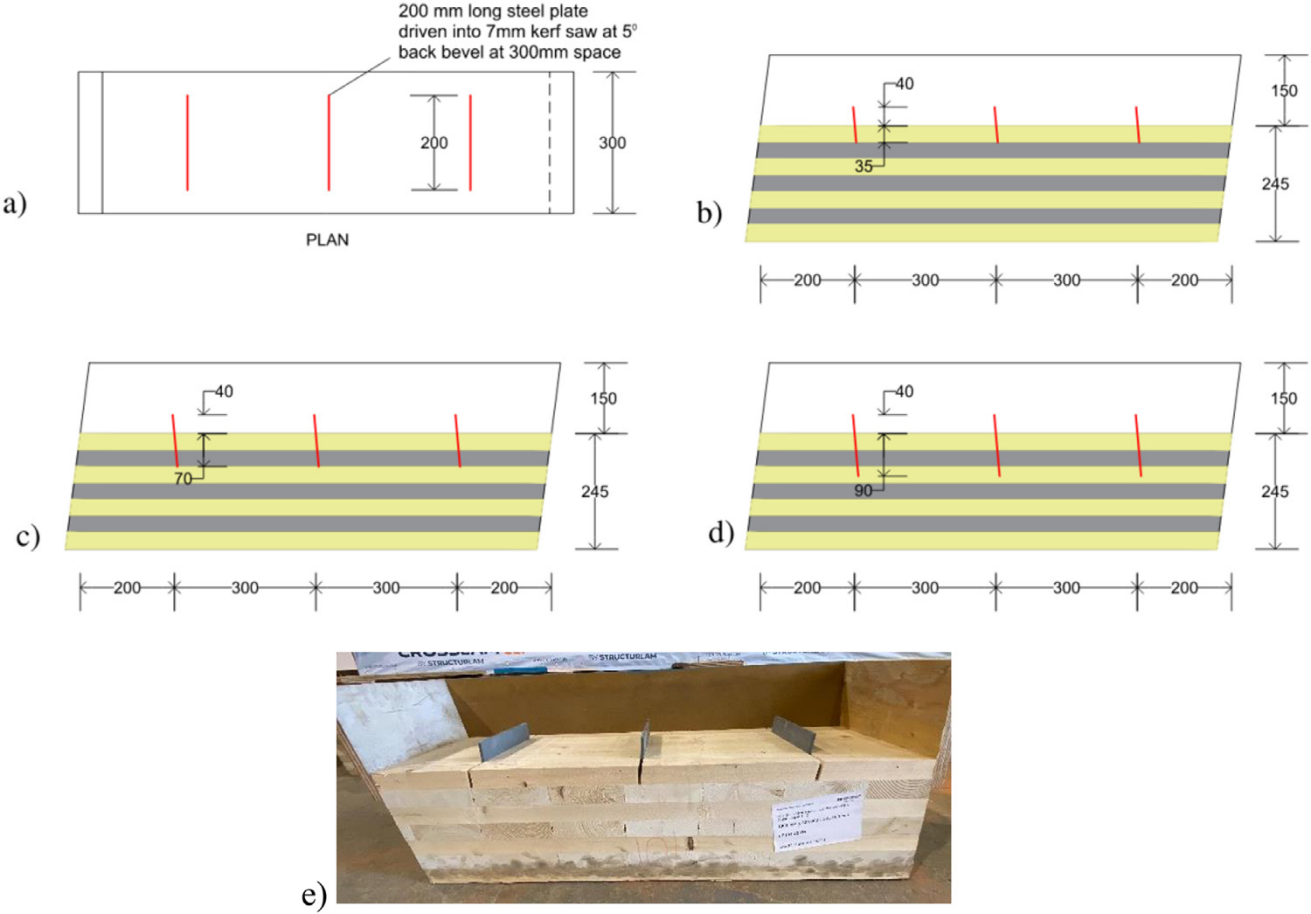
TCC kerf plate connector: (a) plan view with steel plates, (b) S1-A, (c) S1-B, (d) S1-C; (e) photo of a CLT panel with steel plates before concrete pouring.

**Fig. 2:**
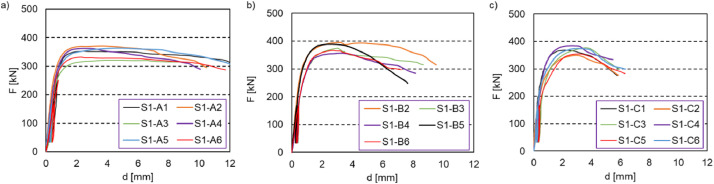
Dataset #1- load-slip curves for small-scale specimens: series – a) S1-A: TCC Type A STS shear connector, b) S1-B: TCC Type B steel kerf plate shear connector, c) S1-C: TCC Type C HBV mesh shear connector.

Dataset #1 supplies the data related to small-scale shear tests on TCC connectors using kerf plates (Fig. 1). The dataset contains relative slips measured along the shear plane between CLT and concrete. From this data, one can obtain the load-slip curves shown in Fig. 2. The test specimens were 1000 mm long and 300 mm wide. From these small-scale tests, it was observed that the kerf plate embedment depth into CLT was the primary factor that affects the connection stiffness and shear strength.

The rationale behind the selection of the specific shear connections and the comparison between various shear connectors was discussed in preceding publications [1,2].

**Fig. 3:**
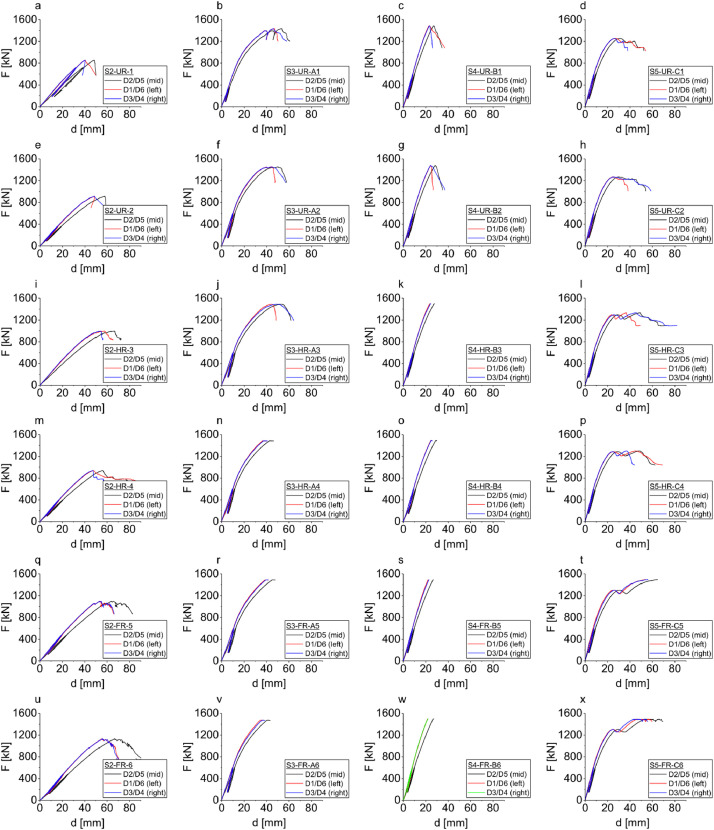
Dataset #2- load-deflection curves for half-scale specimens tested under bending and deflection measured at mid-span using sensors D2/D5, at one-third using sensors D1/D6, and two-third using sensors D3/D4: series S2 CLT only– a) e) unreinforced S2, i) m) low-reinforced S2, q) u) high-reinforced S2; series S3 TCC with Type A STS shear connector – b) f) unreinforced S3, j) n) low-reinforced S3, r) v) high-reinforced S3; series S4 TCC with Type B steel kerf plate shear connector – c) g) unreinforced S4, k) o) low-reinforced S4, s) w) high-reinforced S4; series S5 TCC with Type C HBV mesh shear connector – d) h) unreinforced S5, i) p) low-reinforced S5, t) x) high-reinforced S5.

Dataset #2 provides floor deflections and interface slips of half-scale specimens. The half-scale test specimens were 5030×2400 mm, constituting half the length of the actual floor size used in construction. This dataset contains test series S2 to S5 (Table 2). Series S2 is the bare CLT panels without concrete topping. Series S3, S4, and S5 are TCC floors with Type A, B, and C connectors, respectively. Each test series consists of unreinforced, low- and high-reinforced specimens where screws were widely and closely spaced, respectively. These specimens were intentionally designed for shear failure. Deflection was recorded at mid-span and one-third loading points on both sides of the specimens. From this dataset, one can obtain mid-span load-deflection curves as shown in Fig. 3, interface slips measured between concrete and CLT panels at 8 different locations along the length of the floors on both sides (4 locations on each side), and the warping of the panel’s edges are also listed in this dataset. The half-scale tests showed that the types of shear connectors and the quantity of screw reinforcing are the two primary factors influencing the effective bending stiffness and shear load capacity of the CLT-concrete composite system.

**Fig. 4:**
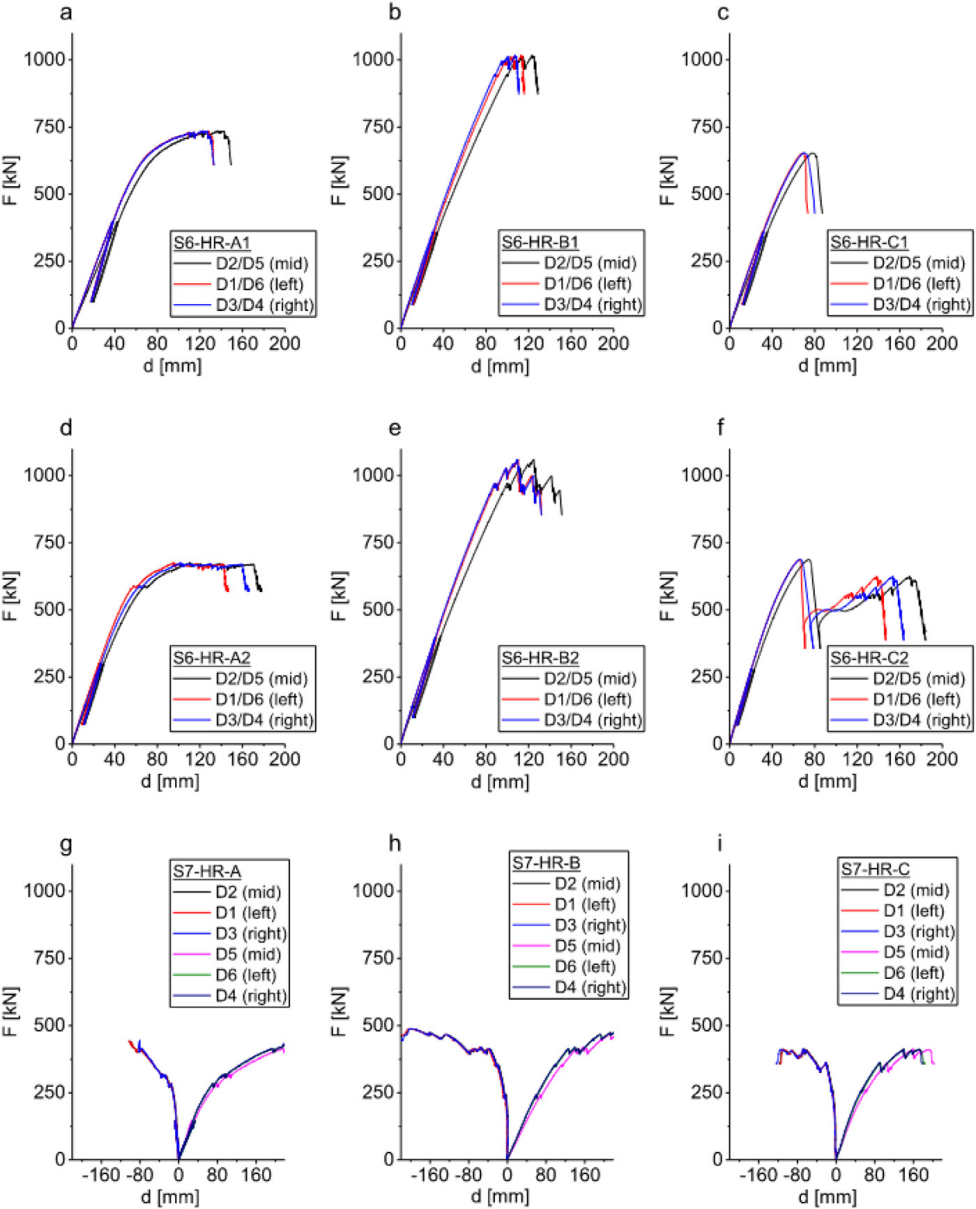
Dataset #3- load-deflection curves for full-scale low-reinforced specimens deflection measured at mid-span using sensors D2/D5, at one-third using sensors D1/D6, and two-third using sensors D3/D4: series S6 tested under bending – a) d) S6 TCC with Type A STS shear connector, b) e) S6 TCC with Type B steel kerf plate shear connector, c) f) S6 TCC with Type B HBV mesh shear connector; series S7 tested under torsion – g) S7 TCC with Type A STS shear connector, h) S7 TCC with Type B steel kerf plate shear connector, i) S7 TCC with Type C HBV mesh shear connector.

Dataset #3 provides floor deflections at three locations, the interface slips between CLT and timber at 8 locations and the panel edge warping at 6 locations for the full-scale specimens. The full-scale test specimens were 9630×2400 mm which was the same length of the actual floor size used in construction. Two series S6 and S7 are listed in this dataset. Both series contained specimens of all three types of connectors; the CLT panels were low-reinforced. From this dataset, one can obtain mid-span load-deflection curves as shown in Fig. 4. The full-scale tests showed that the types of shear connectors is the main factor influencing the effective bending stiffness and bending moment capacity of the full-scale CLT-concrete composite system.

## 4 Experimental design, materials and methods

### 4.1 *Materials*

The TCC floors were comprised of grade E1M5 7-ply, 245 mm thick CLT panels, fabricated by Structurlam at Penticton, BC, is manufactured in accordance with ANSI/APA PRG 320 [4] with SPF (Spruce-Pine-Fir) lumber, 2100 Fb-1.8E machine-stress rated grade for the major strength axis laminations and SPF No.3 grade for the minor strength axis laminations. The relevant material properties satisfied as per CSA O86 requirements [5]. The tested floor specimens were supported on 430 mm × 1178 mm grade 16c-E Douglas Fir glulam columns. A 150 mm concrete topping of 35 MPa minimum specified strength was added on CLT panels. Type I Portland cement was used with a maximum aggregate size of 10 mm and superplasticizer to achieve a high-flow, 80 mm slump. The average concrete cylinder compression strengths from day 7, 28, and 34 were 33 MPa (CoV = 2.8 %), 47 MPa (CoV = 2.7 %) and 50 MPa (CoV = 1.5 %), respectively. The concrete was reinforced with 10 M longitudinal rebar at the top and bottom spaced 150 mm on centre, and 10 M transverse rebar (stirrups) spaced 300 mm on centre.

Three types of connectors were used for the test program: Type A – ø11×250 mm fully threaded STS [6] were installed at an angle of 45°; Type B – grade A36 [7] 6 mm thick and 75 mm deep and 2100 mm long steel kerf plates were installed by hammering them into a 7 mm saw kerf; and Type C – proprietary glued-in perforated plate HBV connector system [8], consisting of 90 mm deep and 2 mm thick perforated plates were glued into a 3 mm kerf using a polyurethane-based adhesive.

### 4.2 *Test overview*

**Fig. 5:**
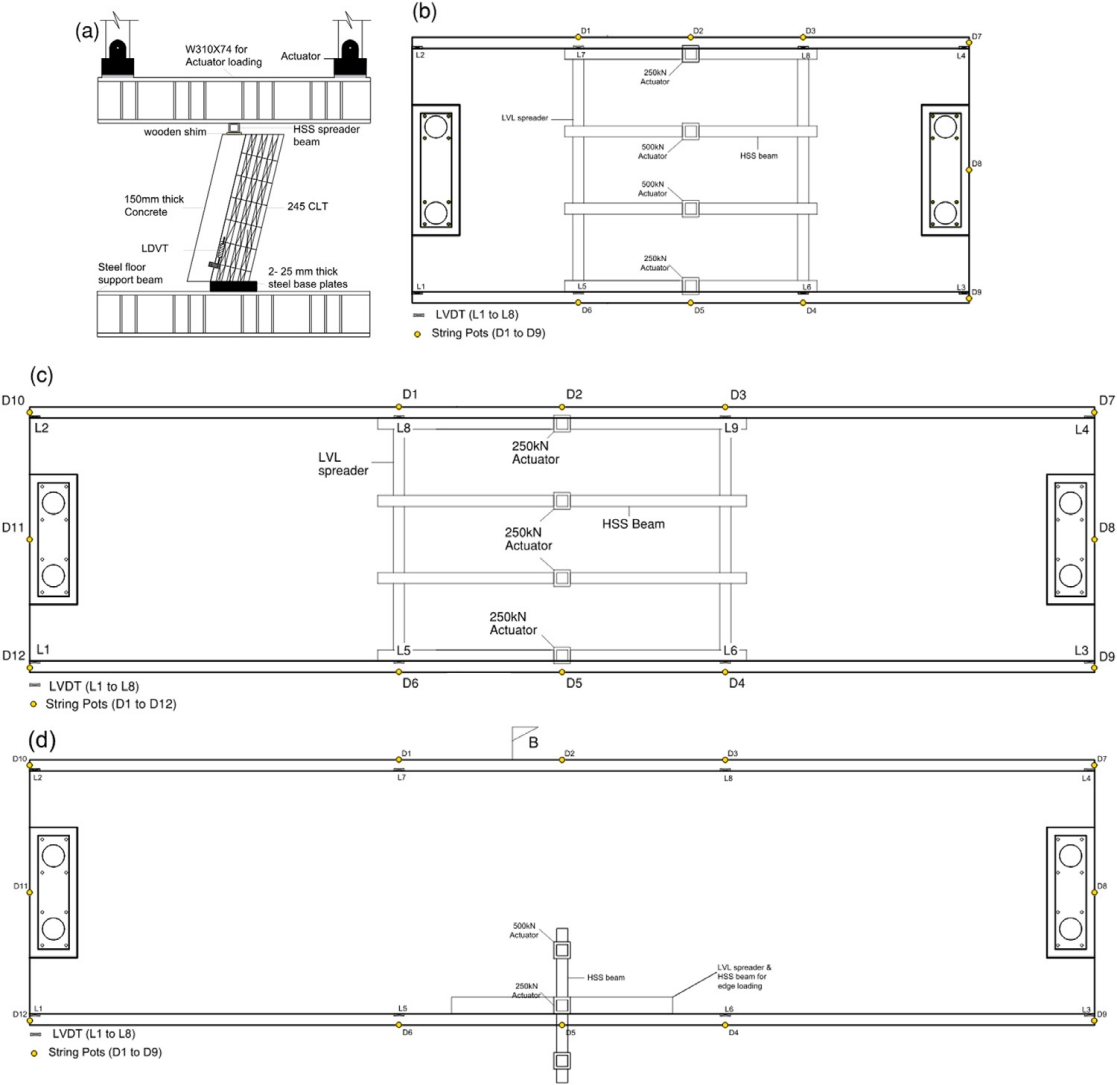
Schematic test setup for a) dataset #1- series S1, b) dataset #2- series S2-S5, and dataset #3- c) series S6, and d) series S7.

**Fig. 6:**
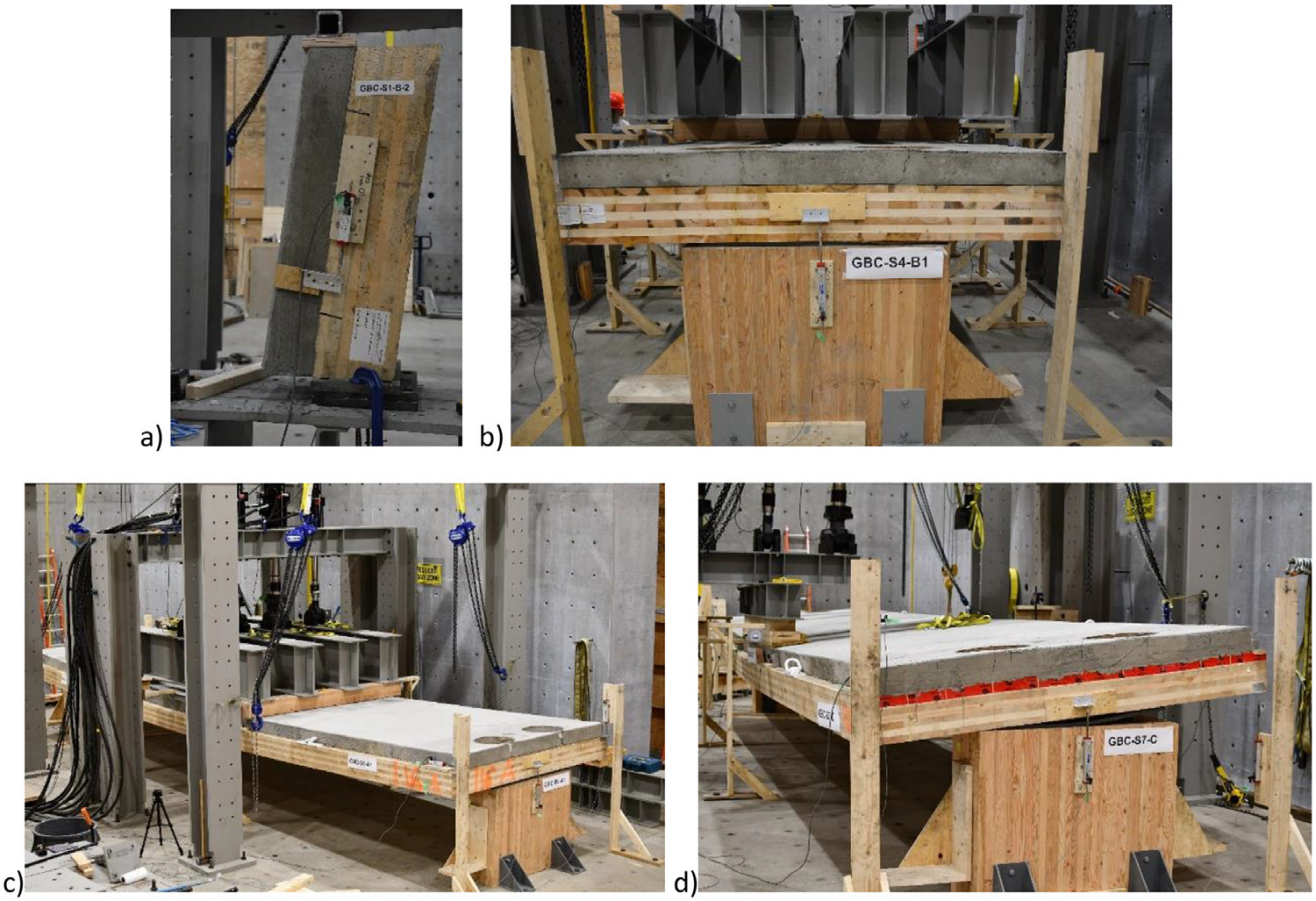
Test photo for a) dataset #1- series S1 (shear test), b) dataset #2- series S2-S5 (4-point bending test), and dataset #3- c) series S6 (4-point bending test), and d) series S7 (torsion test).

Dataset #1- In the small-scale series S1, three subsets were tested under in-plane shear as shown in Fig. 5, Fig. 6. The subsets included three embedment depths into - a) the full first layer of CLT (35 mm), b) the full second layer of CLT (70 mm), and c) the partial third layer of CLT (90 mm). A displacement-controlled load at a constant rate of 5 mm/min was applied.

Dataset #2- The half-scale series S1-S5 were tested under 4-point bending (Fig. 5, Fig. 6) under displacement-controlled load at a constant rate of 15 mm/min. Loads were applied at one-third points using two 500 kN and two 250 kN actuators, totaling a maximum capacity of 1500 kN. These loads were distributed along the third-point using spreader beams. If specimens did not fail after reaching the maximum load, the test setup was changed, and the specimens were re-tested under three-point loading.

Dataset #3- Full-scale series S6 was subjected to 4-point bending loading (Fig. 5, Fig. 6) under displacement-controlled load at a constant rate of 15 mm/min. The floor loads were applied at one-third points using the beforementioned four actuators. Additionally, full-scale series S7 underwent torsional loading with loads applied to one edge. The schematic and test photo for torsional test setup are shown in Fig. 5, Fig. 6, respectively.

## Limitations

Due to limited funding, only two replicates were tested for all mid-scale and full-scale series, except for the full-scale torsional tests, which were tested with only a single replicate. However, this limited number of tests was deemed appropriate for floors tests and is in line with what is commonly done for TCC floors. Further, the specimens were tested under controlled laboratory conditions. The conditions under floors are fabricated on-site may differ. Factors such as moisture management of timber, curing conditions of concrete, and other environmental influences must be carefully managed to ensure that the real-world performance of TCC floors can be expected to be similar to those tested in this study. Finally, despite rigorous efforts to ensure accurate calibration and measurement using a range of sensors, there is an inherent possibility of measurement errors.

## Ethics Statement

The authors assure that the manuscript adheres to ethics in publishing standards. The authors confirm that no experiments were conducted on humans or animals, and the data were not collected from social media.

## Credit Author Statement

**Md Shahnewaz:** Conceptualization, Methodology, Experimental Design, Visualization, Investigation, Writing- Original Draft Preparation. **Robert Jackson**: Conceptualization, Methodology, Experimental Design, Supervision. **Thomas Tannert**: Experimental Design, Experimental Setup Design, Data Curation, Supervision, Writing- Reviewing And Editing.

## Data Availability

Mendeley DataExperimental dataset for cross-laminated timber-concrete composite floor systems (Original data)
